# Niche Cells Crosstalk In Neuroinflammation After Traumatic Brain Injury

**DOI:** 10.7150/ijbs.52169

**Published:** 2021-01-01

**Authors:** Yibin Jia, Guanyi Wang, Yuqing Ye, Enming Kang, Huijun Chen, Zishuo Guo, Xiaosheng He

**Affiliations:** 1 Department of Neurosurgery, Xijing Hospital, Airforce Military Medical University (Fourth Military Medical University), Xi'an 710032, China; 2 Department of Neurosurgery, PLA 163rd Hospital (Second Affiliated Hospital of Hunan Normal University), Changsha 410000, China; 3 State Key Laboratory of Pathogen and Biosecurity, Beijing Institute of Microbiology and Epidemiology, AMMS, Beijing 100071, China

**Keywords:** Traumatic brain injury, Neuroinflammation, Nerve regeneration, Extracellular vesicles, Microglia, Astrocytes, Neural stem cell (NSC)

## Abstract

Traumatic brain injury (TBI) is recognized as the disease with high morbidity and disability around world in spite of the work ongoing in neural protection. Due to heterogeneity among the patients, it's still hard to acquire satisfying achievements in clinic. Neuroinflammation, which exists since primary injury occurs, with elusive duality, appear to be of significance from recovery of injury to neurogenesis. In recent years, studied have revealed that communication in neurogenic niche is more than “cell to cell” communication, and study on NSCs represent it as central role in the progress of neural regeneration. Hence, the neuroinflammation-affecting crosstalk after TBI, and clarifying definitive role of NSCs in the course of regeneration is a promising subject for researchers, for its great potential in overcoming the frustrating status quo in clinic, promoting welfare of TBI patient.

## Introduction

For years, despite decreasing incidence of severe types, traumatic brain injury (TBI) is still the disease that causes the morbidity and mortality around world 1, 2. Canonically, researchers mainly pour energy and captivity into exploring the neural recovery methods, such as medicine or cell treatment to enhance the neurogenesis 3, whereas little achievements were acquired 4. In common, though no delimitation indeed exists, we customarily categorize TBI into “primary injury” and “secondary injury” 5. Initiated with tissue damage and cell death, innate immune cells are activated, releasing “expanding-signal” to recruit other immune components to enlarge the reaction. Observation from the beds proves secondary injury as major reason contributing to the loss of neural cognition and function, with lasting and chronic damage to the brain, such as loss of glutamate homeostasis 6, 7, proteins and phospholipid membranes damaged by free radicals 8, and the inflammatory response comprised of both classic and neurogenic inflammation 9. In addition, absence of qualitied TBI model exactly representing complex clinical situation 10, 11, and the lack of knowledge on elucidating the pattern of the secondary injury, account for poor understanding on treatment and rehabilitation 2.

The neuroinflammation occurs after the primary injury, is thought to be of great significance in either the recovery of injury, or proliferation and differentiation of the NSCs 12, 13. It takes a lot for researchers to figures out the definitive role of inflammation in the neurogenesis or neural regeneration, which turns out to be beneficial in early stage but converts to causing damage and neurodegeneration in the long run. To further complicate matters, simply aims to inhibit the inflammatory response, including activation of immune cells or the release of some chemokine and cytokine, didn't work well in the outcome of the TBI patient 14. Understanding the hiding mechanism of the neuroinflammation, which has been proved to have a double-edged function, and then wielding its helpful role in promoting the neurogenesis, is a promising approach to tackle with this clinical dilemma.

“Niche” was first proposed as the microenvironment sustaining the properties of proliferation and differentiation 15. In TBI, the conception is thought as the region echo to the injury, and then gradually regulate this progress and promote recovery (through extracellular vesicles, microRNAs, et). SVZ (subventricular zone) and SGZ (subgranular zone) show as the most potential place to support the regeneration 16, 17. The aim of treatment on injury comes to recovery of both function and structure, referred as neural regeneration in brain. The component in the neurogenic niche constitute of different cells (neuron, glial cells) and structures, ranging from simple to delicate. Each member has unique but cooperative function to maintain the hemostasis or be activated to make a response to the irritation 18-21. NSCs and its progenitor cell, the cluster have the ability to promote regeneration and repair the damage caused by TBI, is considered as the central players in communication and crosstalk in the regeneration progress 18, 22. Providing an panorama of neurogenic niche activity, may shed a light on modulating neuroinflammation toward to a regeneration-beneficial way, ensued with enhancing neural function and cognition in patients with TBI.

A number of the progress and literature emerging these years, makes it necessary to further summary the inflammation on neurogenesis after the TBI. In this review, we propose a sketch of post-traumatic neuroinflammatory events, and a constellation of resident cells in the niche reacting to the inflammation, promoting recovery and neurogenesis. Following the communication and interaction between the niche cells, involving factors are mentioned. In the ensuing sections, we aim to investigate NSCs as the leading role in the modulation of neurogenesis in the inflammation after TBI.

## Traumatic brain injury and the inflammatory response

To struggle for clear understanding on TBI, even nowadays, there still remain controversaries that if any model could appropriately mimic the complex process in vivo 4, 23. The primary injury of TBI is mainly the injuries about mechanical damage to the tissue, cell membrane and BBB 24. As a heterogeneous entity, TBI comprise a series of mechanical injury patterns such as extrinsic compression from mass lesion, concussion, diffuse axonal injury 25, following a range of pathological mechanisms by which neuronal injury can be aroused, such as ischemia, apoptosis 26, mitochondrial autophagy 27, cortical spreading depression 28, edema 29 and microvascular spasm. The neural inflammation is thought to company with these courses all along, and the long-lasting inflammatory response seems to link the TBI with other neurodegenerative disease (AD, PD, CTE) 30.

The inflammatory response in neural system is a sophisticated, complex composition of processes including the ignition of the sensor, signals transducing, activation of the immune cells and at last, continuous inflammation. As a paradigm 2, at the time of injury happens, the alert signals (ATP, HSP, HGMB1, bradykinin) was released into neural system, which are generally considered as PAMP or DAMP, binding to the receptors on the sensor cells 31-33. Pattern recognition receptors (PRRs), like Toll-like receptors (TLR), receptors of advanced glycosylation end-products (RAGE) or purinergic receptors that are commonly expressed on the innate immune cells to trigger the downstream reactivity facilitated by the immune active molecules 34. It is established that some receptors and signals are associated with the outcome of TBI patient, on the evidence that blockade or knockout those can effectively reduce cerebral edema, lesion volume and the release of inflammatory factors like IL-1β, IL-6 35, 36.

Normally, the inflammation in the neural system can be classified into “classical inflammation”, which immune response reacts just as other tissue injury (the secretion of the substance P) with plot similar with periphery, and “neural inflammation”, (involving the cells and molecules exclusive in the brain) 14, 37. The damaged tissue or innate immune cells are the first to react to the injury, then by secreting chemokines and cytokines, the system can in turn recruit the glia cells, T cells to aggravate at the injured site. Consequently, the inflammation is further expanded and augmented, and the resident cells, like microglia (MG) or astrocyte (AG), neuron would make a difference in inflammation and neurogenesis 20, 38, in cooperation with other immune cells from periphery.

The inflammation response doesn't play a clarified role in neurogenesis, for existing evidence are unable to come to a definite outcome 39. Inhibition or blockade of the releasing of the cytokines like IL-6, IL-1α and TNFα, seems to have beneficial results in the inactivation of the immune cells and the enlargement of immune response, while has little improvement in clinic outcome 40. Similarly, depleting the neutrophil in mice effectively attenuates the immune injuries like edema or tissue loss, accompanied by reducing MG activation, but with a poor function recovery 41. And using minocycline was found able to reduces chronic MG activation, but with an increasing neurodegeneration and aggravated cognitive deficits 42, 43. It seems that in starting episode, there exists a mixture component of “pro” or “anti” inflammatory, and more “pro” in long term 44. Anyhow, more convincing evidence and finding are urged to illustrate this dualism in the inflammation.

## Neurogenic niche cells in neuroinflammation

### Microglia

As the "macrophages" of the central nervous system, MG accounts for about 5-15% of all brain cells 45. Unlike macrophages in non-nerve tissues, MG originates from the yolk sac in the embryonic stage, expressing the transcription factor RUNX1, and the tyrosine kinase receptor c-kit, developing from the red-myeloid precursor cells through the transcription factor Pu. 1- and Irf8-dependent pathway 46. MG is highly branched, sensitive to the changes in neural environment, constantly detecting changes or external stimuli in surrounding environment, responding quickly, receiving, transducing and integrating corresponding signals to produce a series of biochemical activities to maintain the steady state of the internal environment 47. At the same time, with changes in various conditions (aging, injury, disease, etc.), MG will also show new phenotypic characteristics 48. It's believed that activation of microglial receptor involves not only changes in morphology or movement, but also changes in neurotransmitter release, modulation of neuronal-glial synaptic transmission, and cytokines secretion, generation of ROS. Especially, these changes are further involved in the process of secondary injury. The different types and severity of TBI, through the activation of the pertinent receptors, will also make the activation of MG follow different patterns, thereby showing the pro-inflammatory or anti-inflammatory state at different moments 49.

Traditionally, at the time inflammation fires, MG in resting state will be induced by different environments or factors, and "proinflammatory" or "anti-inflammatory" effects will appear (For example, stimulated by LPS, IFN-γ, the cell shows M1 type, and after IL-4 and IL-13 stimulation, it tends to represent as M2 type) 50. Usually, M1 will participate in the secretion of pro-inflammatory factors (TNF-α, IL-6, et), aggravating inflammatory response of the environment, while M2 type may secrete protective, anti-inflammatory factors such as IL-10 to regulate the inactivation of pro-inflammatory cell phenotype, thereby maintaining the homeostasis of the environment 49. Nevertheless, "polarization" in MG is not an issue on absolute conversion of phenotypes, but a relative status that overlaps in time and space. In recent years, with the endeavor in neuroinflammation research, the study on MG has become more comprehensive, and more scholars have advocated abandoning “M1/M2 typing” to represent the function state of MG in the study, but combining the technical knowledge of transcriptomics, proteomics, and single cell sequencing to dynamically observe or describe the changes of MG throughout the inflammatory response.

### Astrocytes

With findings in these years, AG, just like MG, are thought to make a significant contribution to immune response after TBI 51, 52. Apart from the functions in homoeostasis, such as secreting active factors, supporting the neuron and the uptake of Ca2+, AG also have unique role in neural inflammation 53. Following TBI, the cells are activated by mechanical damage, for they are sensitive to the physical stretch or pressure, enriched with corresponding mechanosensitive ion channels. Then, partly like injury tissue, some alert signals are released from the cells. Particularly, along with the calcium dysfunction 54, ATP participates in the cascade amplification reaction, that is, response in activation and recruitment of reactive AG, as well as other immune members (MG, neutrophil, et). In the meantime, with reaction to the injury, other factors also be secreted, like MMP-9, endothelin-1, and isoprostanes, which as well take part in the repairing or maintain the structures and functions in the brain. It is well known that AG is of importance in the integrity of the BBB 55, 56, the excitotoxicity aroused by glutamate, and the formation of glial scar, while just like the dualism of the inflammation, the reactivity of the AG also presents a double-edged effect 53, 57.

Reactive astrogliosis is the consequence of the response to TBI, with the changing in the morphology and the ethology of the AG, which is found to be both protective and harmful in the regeneration of the neural system. AG not only produce alarmins or DAMP (HMGB1, HSP, S100 proteins), interestingly, similar with the MG, but also have the TLR4 and RAGE to amplify neuroinflammation, inducing NF-κB transducing and resulting in the release of inflammatory factors such as cyclooxygenase-2, TNFα, connective tissue growth factors (CTGF), and MMP-9 58. It is also found that NF-κB can trigger cell swelling and edema associated with TBI 59, probably influenced by the dysfunction of the AQP1 rather than AQP4 60. In addition, in Sox2-deletion mice, markedly ameliorated injury-induced tissue loss and behavioral deficits was observed 61, which indicates the correlation between neuroinflammation and other pathologic response, is far beyond illustrating. Notably, the secretion of these initial signals hides the silver lining of the inflammation in TBI, for their can simultaneously aggravate inflammatory impairment or restore neural microenvironment.

### Neurons

Neurons are the fundamental constituents and basic functional unit in CNS. As fragile and delicate, even the mild trauma can lead to neuron loss and axonal injury 62. As the main “victim” in TBI, neurons experience a series of pathological course which ultimately result in the enlargement of the neuroinflammation. The death of the neurons is accompanied with the degeneration of the associated axons, the two both act as powder-hose in the initiation of the neuroinflammation. In closed-head TBI model, the activation of the MG is localized at the region with injured neurons, which starts as the central of the inflammation 63.

TBI leads to series of profiles changes in neuron to present protective or harmful effects, initiating consequential inflammation and oxidative stress. For instance, induction of nociceptin/orphanin FQ 64, accounts for the pain, anxiety and even inflammation in rat model 65, and down-regulating IRF6 is proven to attenuate neuronal apoptosis 66. The key to enhance the survival of new-born neurons from neurogenic niche, is lessening aberrant development and ectopic localization 67.

### Oligodendrocytes

The study on the oligodendrocytes (OLs) in the inflammation after TBI is limited, in spite of the existed evidence that the cells are involved in the chronic demyelinating diseases. Different from the precursor cells (OPCs), those who have the ability in proliferation and differentiation, OLs is mature, non-differentiated, and sensitive to the excitotoxicity 68. After traumatic axon injury, the OLs are prone to demyelination, along with the loss of action potential 69. The loss of myelination, resulted from primary injury or secondary injury, may be accompanied with the loss of the axons, and induces OLs death end up with caspase 3-mediated apoptosis 70, 71. Afterwards, axonal regeneration is inhibited by myelin debris and MG is activate as a further trigger of chronic neuroinflammation 44, 72, 73, resulting in neurodegeneration and cognitive impairment at last.

**Figure 1:**
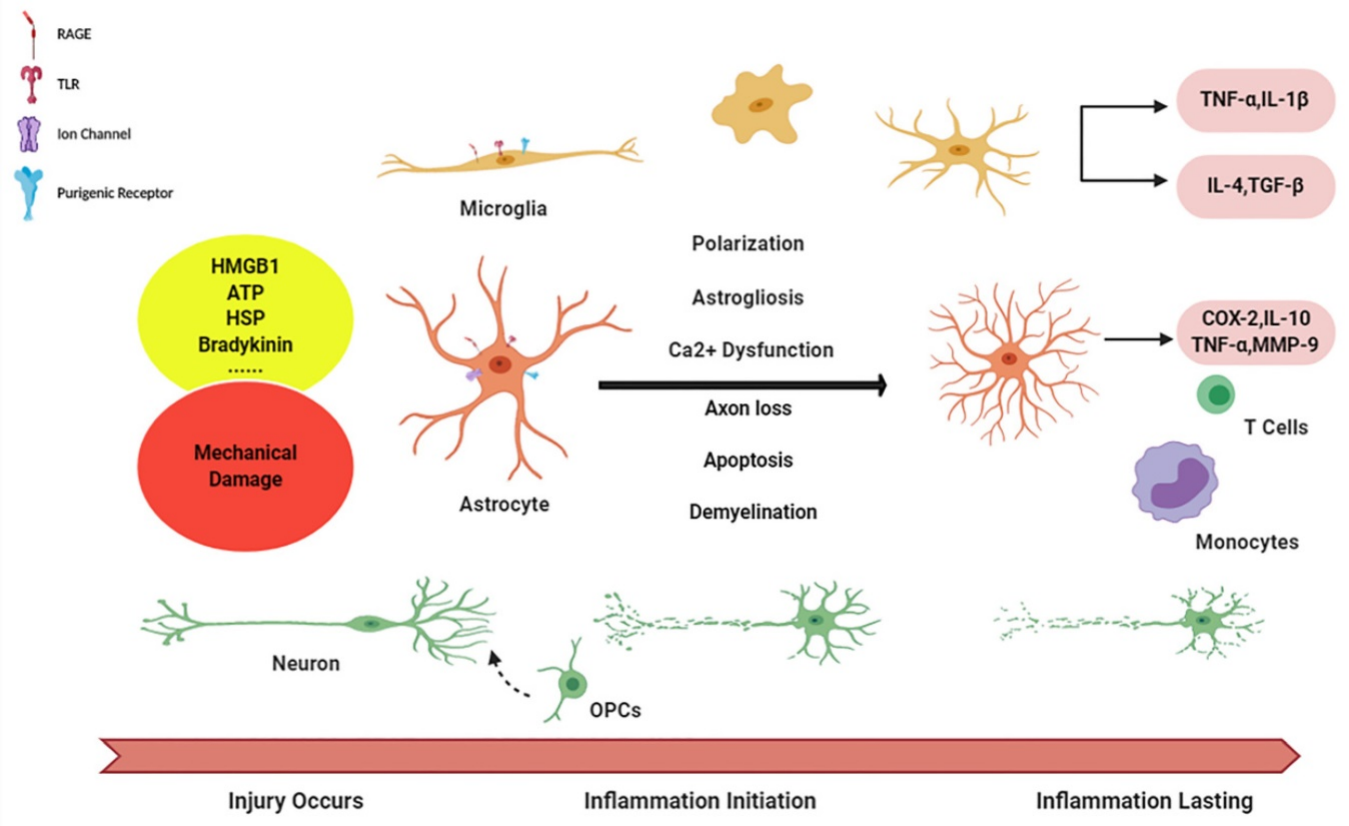
** Niche cells in initiation and aggravation of neuroinflammation.** Diagram succinctly shows the course of neuroinflammation after TBI. The alarmin signals are released from damage tissue and, via activating corresponding receptors on glia cells, initiating microglia, as well astrocytes activation, followed with the cascade in enlargement of immune response with inflammatory factors releasing. And reciprocally, inflammation becomes chronic, long-lasting condition in during the crosstalk (function of peripheral immune cells has not been illustrated in this figure).

OPCs are characterized with PDGFRα and NG2, namely NG2+ cells, with various functions in CNS like morphological changes, axons protection and glia scar formation 74. It is also found that, after TBI, OPCs express GFAP and plausibly have ability to differentiate into AG 75. As reaction to damage, an increasing amount of OPCs gather in the injured region 70, 76, regulated by NG2, growth factors and glutamate 77, 78. Myelination might be of importance in plasticity and recovery of function after TBI 79. However, the role of OPCs in inflammation is ambiguous, for their interaction with vessel seems to disrupt the BBB and exacerbate neuroinflammation in MS 80 (Figure 1).

## Communication, crosstalk and integration in neuroinflammation

### Crosstalk among cells in inflammation and neural regeneration

TBI changes the reaction pattern in neurogenic niche, activating unique cell interaction in post-injury neurogenic response. The relationship between these niche cells tends to be more complex, though rather novel molecules emerge. It's likely that these findings in cells and molecule are weaving signal network to present the series on neuroinflammation after TBI.

For the controversy above, in recent years, researchers incline to categorize the AG into two groups, A1 and A2, with different phenotype, which resemble MG. A1 characterized as the upregulation of C3 and can be harmful for the neurogenesis. Especially, it is elicited by the reactive MG that secret lL-1α, TNF and C1q, leading to the death of neurons and OLs 81. On the contrary, IL-10 originated from AG change the phenotype of MG, as well as lymphocyte density, to improve the neural survival 82. The crosstalk between MG and AG after TBI is critical in the persistent neuroinflammation 51. AG and OLs can produce IL-33 as the alarmins to promote the aggregation of the MG, followed by an altered cytokine/chemokine profile 83.

Using CSF1R antagonism (PLX5622) to eliminate the MG, which is activated by injured neuron, can attenuates rod microglial formation and astrogliosis 84. And via downregulation of the purinergic receptor P2Y1, microglia could facilitate AG with scar formation, to behave as a neuroprotective phenotype 85. After the depletion of the NG2, it comes with enhanced astrogliosis and up-regulated anti-inflammatory M2 biomarker Arg-1, followed with decreasing total number of microglia/macrophages, whereas worsening outcome was observed 86, then implicating the complicated mechanism in the neuroinflammation.

The crosstalk among the cells need the participation of specific molecule, like thyroid hormone (T3) might promote the neurogenesis through NSCs indirectly, probably with the neurons 87.

EVs, or specifically referred as exosomes, constituting diverse cargo like RNA, protein or lipids, capable of transmitting contents through BBB, appear to be the most effective courier in the neuroinflammation and nerve regeneration 21, 88, 89. Neurons can generate exosomes enriched with miR-21-5p to regulate the MG towards to M1 type 90. Correspondingly, glia EVs tend to modulate synaptic plasticity via miR-146a-5p 91, even with the sphingolipid on the membrane, leading to the excitatory transmission increase in neuron 92. As the media in the crosstalk between MG and NSCs, exosomes are of key influence on repair of injury 93, and increasing microRNA are explored to regulate the polarization status of MG, further switching the direction of the neuroinflammation 94-97 (Table 1).

### NSC might integrate the inflammatory signal in neural regenerative microenvironment

As the all-around player in CNS, NSCs and its progenitor (NPCs) play a pivotal role in the communication between different cells with high plasticity 18, 103, In the phase after TBI, different molecules, cytokines, chemokines, metabolites and neurotrophic factors from injured site or regulating cell, play roles in the differentiation and proliferation of the NSCs 22, requiring an exquisite cooperation. Observation on cytokine responses after TBI suggest that certain factors induced inflammation may function in an indirect way 104, that is to say, some intermediary exists. Just as transplantation of NSCs can generate OLs promote myelination 105, NSCs exhibit potential in regulating or remodeling microenvironment. Depletion of the transcriptional regulator Id3 leads to a decreasing number of AG generated from SVZ NSPCs and Id3(-/-) adult NSPCs are unable to differentiate into BMP-2-induced AG. While NSPCs deficient for transcription factor E47, which is found to be downregulated by Id3, shows performance of differentiation again, in the absence of BMP-2 106, indicating a balance in the differentiation course of NSCs. Neonatal AG also promote the proliferation of NSCs with secreted protein 107. Represented with similar function, NPCs secrete factors like TGF-β2 to transform MG into protective type 102, who in turn to support the maintenance and growth. The reparation on demyelination can also be initiated by M2 microglia, while through NSCs to bring about oligodendrogenesis and myelination 108.

Accumulating evidence has demonstrated that NSCs niches is the utmost region as the origin of the neural regenerative microenvironment, though its program of neurogenesis is a bit distinguished from the pattern after TBI. Apart from well accepted SGZ and SVZ, some new regions need more evidence to play a role in neurogenesis after TBI 109, 110. And intriguingly, these regions seem to show response to the TBI rather than SCI 111, probably, on account of the different signals in blood.

BBB might connect the crosstalk between periphery and CNS, the collapse of BBB accounts for lasting neurodegeneration disease 112. The degeneration of the ECM leads to the disruption of the BBB, and ECM-remodeling transcriptional changes can be induced by the serum protein albumin via TGFβ signaling in primary AG 113, 114.

**Figure 2:**
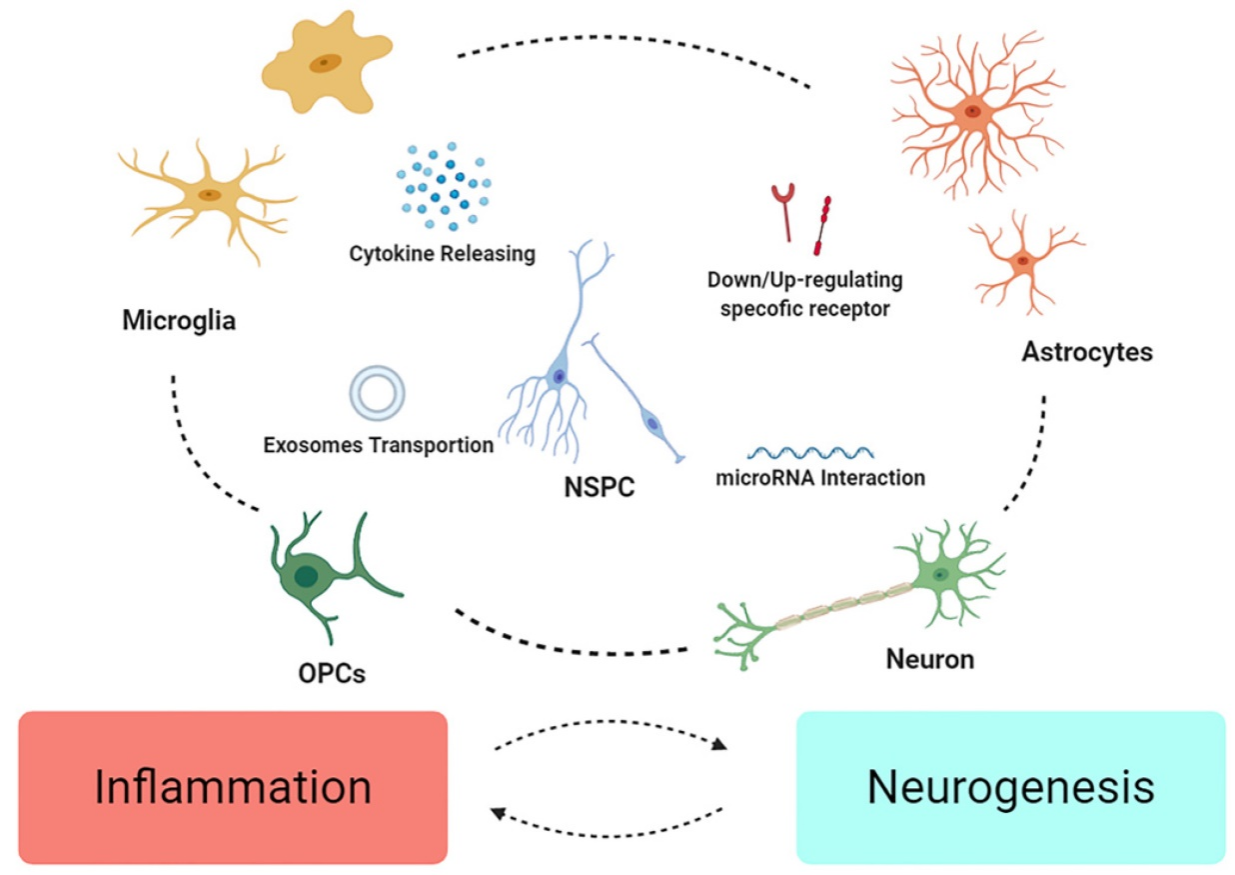
** NSCs might integrate the inflammatory signals crosstalk in neuroinflammation.** Different cell may down/up-regulate specific molecule/receptors to enhance or weaken others. Microglia and astrocytes might mutually influence their phenotype as “pro” or “anti” effect on inflammation, and change the fate of neurons and OPCs. Finally, NSPC might act as intermediary among various crosstalk, modulating signals, restructuring microenvironment, and further promote recovery from the injury.

NPCs may be of particular influence in secondary cytokine releasing 104, in response to the simultaneous inflammatory factors. Researchers firstly found that acute inflammation may induce neuronal regeneration more than damage in zebrafish CNS 115, coupled with unique role of Gata3 116. It is notable that proliferation of NSCs coincide with the expression of TLR4 117, which can be conjectured the subtle connection between neurogenesis and inflammation, attracting the interest in inflammation regulation after TBI, like NSCs regulating NLRP3 and IL-1β, hence attenuating the neurotoxic cascade induced by MG 118. Otherwise, after transplantation, NSC/NPCs tend to alter phenotype of MG and ameliorate inflammation 105, 119, that betokens EVs from NSCs also show anti-apoptotic and anti-inflammatory properties 120. This might attribute to the role of inflammation on spatiotemporal reason. It's reasoned to speculate that immune response only assumes beneficial in the primary injured part (Figure 2).

## The promising method for regulation of neuroinflammation and enhance the neurogenesis

As promising treatments, those focused on the regulation of the inflammation, metabolism or the neurogenesis are emerging, it is worth believing TBI patients can acquire better outcome and welfare.

Existing evidence has proposed potential method to regulate the inflammation. knockdown of TLR4 can ameliorate neuroinflammation after TBI, in a way by inhibiting autophagy and AG activation 121, and salsalate, an unacetylated salicylate, is utilized to relieve inflammation and promote function recovery 122. Downregulation of the inflammasomes is also a potential method to attenuate the damage caused by inflammation response 123.

New target for the treatment of TBI has been aroused, such as CCR5 124. And some dynamic biomarker, like Nrf2 125, may be useful to evaluate the process TBI came through. Combined using minocycline and N-acetylcysteine, inducing remyelination and regulating neuroinflammation, seems superior to the monotherapy attempts before 126, 127.

Apart from the regulation of the inflammation, methods are exploited to improve the oxygenation or metabolism status, and weaken neuronal pyroptosis 128. Hyperbaric oxygen therapy and Hydrogen gas both seem to be effective to attenuate the injury extent 129. Lactate metabolism in brain is an essential feature after TBI 130, and hypertonic sodium lactate solution is proved to reverse brain oxygenation and metabolism dysfunction after traumatic brain injury through vasodilatory, mitochondrial, and anti-edema effects 131.

Stem cell therapy, for its multi-differentiated capability, is a promising method 132, 133. However, though application of iPSC, is still limited for ethical issues or bio-safety. MSCs, which is thought to be the most optimum candidate to treat the neurodegeneration disease, no matter injecting cells directly or the extracellular vesicles (exosomes) 89, 134, striking improvement in both neurogenesis or recovery of the function have been acquired 135, 136. Furthermore, a host of ncRNA are involved in the TBI diagnosis and treatment 137.

## Conclusion and future perspectives

TBI is still the disease affecting long-term quality of life with protracted course of neuroinflammation 1. The types of it, though, differ in pathology and outcome, the long-last and annoying course of inflammation seems to be a common situation. In spite of substantial evidence showing that reactivity in cell lineage is unable to imitate the exact procedures ongoing in vivo, coupled with sufficient microenvironment factors, it's still reasonable to make most use of the established model 10, for direct or indirect regulation in promising treatments. And it's worth celebrating that certain attempts seems able to balance the function of inflammation in neurogenesis.

To have an explicit vision of neuroinflammation in neurogenesis and nerve regeneration, detailed work and evidence are needed 2, 14, 39. Evidence available is unable to clarify definitive role of inflammation after TBI, in view of complicated and mixing essence itself. Existing evidence shows feature as “protective” in acute inflammation and “harmful” in long term 13, while absence of particular point in time makes it harder to make utmost of advantages in neuroprotection and promotion in regeneration. Switching the polarization status of the MG may be potential, and increasing number of drugs or molecules have been found to be effective 138-140. It shall bear in mind that the concept in polarization is probably appropriate in modulation on inflammatory outcome rather than an absolute condition. More study on multi-dimensional and comprehensive in these immune cells, with burgeoning progress in single-cell technology and integrated perception in CNS 18, 39, 141.

Besides MG, other niche cells represent peculiar functions in initiation, enlargement, or modulation of the neuroinflammation. AG and MG both serve as “mixed bag” 38. Based on the double function on transforming immune response, the crosstalk, or glia-crosstalk, makes it complex to understand the mechanism underlying. Cytokine, chemokine and recently, exosomes are all participating in this interplay, depicting a more panoramic network in this long-studied subject.

Despite dwindling since the neonatal 142, NSCs and its progenitor cells are capable of repairing injury via differentiation, proliferation or replacement. A host of work on the stem cells transplantation in TBI or SCI have shown gratifying results, whereas the translation and clinical application is limited for frustrating ethical or bio-safety issues. Exosomes, by virtue of its various contents and excellent affinity with nervous system, may reveal a better therapeutic potential 19, 88. Acquiring a more comprehensive awareness on the relation between neuroinflammation and neurogenic niche is essential, in the premise that glia, neuron and NSCs all engage in the communication and crosstalk.

Inflammation in neural regeneration is a commonplace, that being said, with more intriguing achievements in these years, it's requisite to balance rather than simply inhibiting it. It is important to reiterate that, among glias, MG are the most potential candidates at present, for its role in magnifying inflammatory response, and AG, based on its affluent secretion property, is a promising participant as well. We didn't refer to ECM, and vascular endothelial in this interplay, those both are of particular importance in pathophysiology. What's more, NSCs, shows fundamentality in the course all long. Clarifying its activity in integrating all the signals, as well as functional signals in promoting proliferation, survival, and migration, is of high-priority. It's believable enough that we are laying the key-stone for the avenue towards clinic translation and the welfare of TBI patients.

## Figures and Tables

**Table 1:** Effector molecules among niche cells in neuroinflammation crosstalk after TBI

Cell Type	Molecules	Effect	Ref
Microglia	miR-146a-5p	Suppresses Syt1 and Nlg1 expression in receiving neurons; leading to dendritic spine loss as well as a decrease in the density and strength of excitatory synapses	91
	IL-6, IL-1β,TNF-α	Enhance reactive astrogliosis and transformation the cells into neuroprotective type	85
	IL-1β, miR-155	Activate additional microglia, progressive immune response in CNS	98
	sphingosine	Modulate synaptic plasticity	92
Astrocytes	miR-873a-5p	Inhibit NF-κB signaling pathway and attenuate neuroinflammation mediated by microglia	95
	GJA1-20 k	Downregulated the apoptosis rate and upregulated mitochondrial function to promote neuronal recovery	99
	IL-10	Ameliorate microglial response and lymphocyte recruitment, promote neuronal survival.	82
Oligodendrocytes	IL-33	Aggravation of microglia/macrophages	83
Neuron	miR-21-5p	Promote polarization of M1 microglia	90
Mononuclear Phagocyte	succinate	Activate succinate receptor 1 (SUCNR1)/GPR91 and represent with anti-inflammatory effects	100
NSC/NPC	IFN-γ	Activate Stat1 Signaling in Target Cells	101
	TGF-β2	Reprogramming infiltrating monocyte-derived cells into anti-inflammatory type	102
